# Hypomethylation of HLA-DRB1 and its clinical significance in psoriasis

**DOI:** 10.18632/oncotarget.12468

**Published:** 2016-10-04

**Authors:** Wenkai Zong, Yiping Ge, Yue Han, Xueyuan Yang, Qi Li, Min Chen

**Affiliations:** ^1^ Jiangsu Key Laboratory of Molecular Biology for Skin Diseases and STIs, Institute of Dermatology, Chinese Academy of Medical Sciences and Peking Union Medical College, Nanjing, China

**Keywords:** psoriasis, HLA-DRB1, gene methylation, mRNA

## Abstract

Increasing evidences indicate that the abnormal DNA methylation is involved in the pathogenesis of psoriasis. A number of SNPs in HLA-DRB1 have been found being associated with the risk of psoriasis, however it is unclear that metylation status within HLA-DRB1 in psoriasis. Here, DNA and RNA were obtained from epidermis of 56 patients with plaque psoriasis and 28 healthy volunteers served as the control group. For the first time, we discovered mean methylation rate for HLA-DRB1 is 52.2%, 64.3% and 68.1% in epidermis from psoriatic lesions, psoriatic non-lesions and healthy controls, respectively. HLA-DRB1 methylation in psoriatic lesions is significantly lower than in psoriatic non-lesions (*t* = 13.077, *p* < 0.001). However, there is no significant difference for HLA-DRB1 methylation between in psoriatic non-lesions and in healthy controls (*t* = 1.046, *p* = 0.299). HLA-DRB1 methylation in psoriatic lesions is negatively correlated to PASI score (*r* = -0.431, *p* = 0.001). HLA-DRB1 methylation in psoriatic lesions of the patients with onset age=18 years is significantly lower than the other patients (*t* = 3.968, *p* < 0.001). Meanwhile, HLA-DRB1 mRNA expression is significantly increased in psoriatic lesions comparing to psoriatic non-lesions (*t* = 12.119, *p* < 0.001). There are no significant difference for HLA-DRB1 mRNA expression between in psoriatic non-lesions and in healthy controls (*t* = 1.172, *p* = 0,245). Moreover, HLA-DRB1 mRNA expression is negatively associated with HLA-DRB1 methylation in psoriatic lesions (*r* = 0.932, *p* < 0.001). In conclusions, our results showed hypomethylation of HLA-DRB1 is associated with HLA-DRB1 mRNA expression and severity of the disease, indicating that hypomethylation of HLA-DRB1 may play roles in the pathogenesis of psoriasis.

## INTRODUCTION

Psoriasis is a common chronic skin disorder that affected about 1%-3% of the world population, and the pathogenesis of psoriasis is still unclear. Based on current findings, psoriasis is generally considered as an autoimmune disease caused by T cell-mediated hyperproliferation of the keratinocytes [1]. Increasing evidences indicate that psoriasis is also a genetic disease triggered by multiple environmental factors, such as smoking, chronic infections, stress, climate changes. Many studies about the association between human leukocyte antigens (HLA) and psoriasis have been reported for a long time [2, 3]. In recent studies, much attention have been paid on the role of epigenetic changes (e.g. DNA methylation) in psoriasis, and the abnormal epigenetic modification of several molecules has been observed in the psoriatic lesions of patients [4, 5]; however, researchers still need to make greater efforts to illustrate the mechanism of how the aberrant epigenetic modifications would affect the pathogenesis of psoriasis.

Human leukocyte antigen class (HLA)-DRB1 is a transmembrane heterodimer cell surface receptor with α and β chains, and each contains extracellular domains, membrane-spanning domain and a tail anchored in the cytoplasm. The α chain of HLA-DRB1 lack polymorphism, and the β chain is encoded by 4 loci DRB1, DRB3, DRB4 and DRB5 [6]. HLA-DRB1 is a major histocompatibility complex (MHC) class II protein that expressed constitutively by antigen-presenting cells (APCs), such as dendritic cells, macrophages etc. The expression of HLA-DRB1 is important for APCs, because HLA-DRB1 can determine the efficiency of APCs to present antigen to T cells [7].

Several studies have examined the relationship between HLA-DRB1 and cancer, including parotid cancer [8] and ovarian cancer [9]. Previous studies suggested that immunohistochemically detectable HLA-DRB1 expression is associated with better prognosis in patients with colorectal cancer [10, 11, 12]. The relationship between HLA-DRB1 and AIDs has also been reported. The level of expression of HLA-DRB1 alleles have been associated with the development of systemic lupus erythematosus (SLE), rheumatoid arthritis (RA), systemic sclerosis (SSc) and multiple sclerosis (MS) [13].

Although the HLA class II locus is the strong genetic risk factor for RA, the relationship between HLA class II alleles and lymphocyte activation remains unclear. The presence of HLA-DR was not confined to activated CD4+ and CD8+ T cells [14]. Nagafuchi et al. [15] studied immunophenotyping of peripheral blood mononuclear cells on RA patients with HLA-DRB1genotype and found the frequency of memory CXCR4+CD4+ T cells was significantly related to severity of disease. A significantly higher frequency of memory CXCR4+CD4+ T cells was found in the patients with one or more susceptible HLA-DR haplotypes, moreover, on B cells, the frequency of memory CXCR4+CD4+ T cells significantly was associated with the HLA-DR expression, which suggested that the interaction between HLA-DR and T cell receptors can regulate the memory CXCR4+CD4+ T cells.

In psoriasis, the role of HLA-DRB1 has also been well investigated. As early as 1982, VB Morhenn et al first observed the abnormal expression of HLA-DRB1 in skin samples of patients with psoriasis [16]. Since then, the polymorphism of HLA-DRB1 and its relationship with the psoriasis, as well as the role of HLA-DRB1 alleles in the pathogensis of psoriasis have been reported in different works [17, 18, 19, 20]. The functional role of the HLA-DRB1 molecule in psoriasis remains enigmatic. In psoriasis, Keratin 17 is a major target antigen of autoreactive T cells. On Keratin 17, the epitopes S1 (118-132), S2 (169-183), S4 (323-337) and S4 (348-362) of HLA-DRB1*04, *07-restricted T cell epitope regions are immunodominant HLA-DRB1-restricted T cell epitopes [21]. Magalhães et al [22] demonstrate these polymorphisms of HLA class I and II may be genetic risk for severity of the disease in a case-control study of patients with mild or severe psoriasis, and found that alleles HLA-DRB1*07, B*37, Cw*06 and Cw*12 were correlated with severe disease course.

However, to our knowledge, the methylation status of HLA-DRB1 in psoratic patients has not yet been investigated. In our previous work, we already proved that hypermethylation of HLA-C may play an important role in psoriasis [23]. In this study, we will focus on the methylation status of HLA-DRB1 in psoriasis and its relationship with the disease.

## RESULTS

### HLA-DRB1 hypomethylation

**Figure 1:**
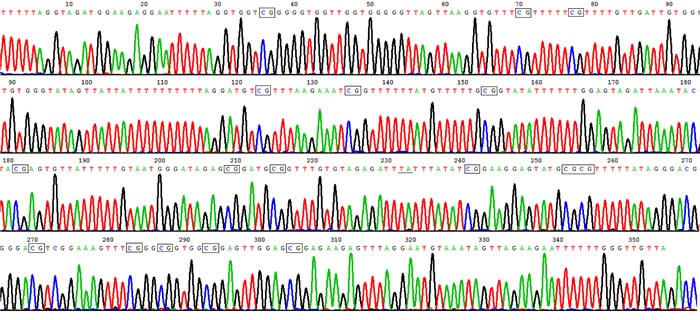
High methylation rate in HLA-DRB1 promoter in epidermis of healthy controls Representative product of sodium bisulfite genomic sequencing showed HLA-DRB1 methylation occurred in 17 out of 18 CpG sites in healthy controls. The rectangles show methylated CpG, and the underline show unmethylated CpG where C change to T after bisulphate treatment.

**Figure 2:**
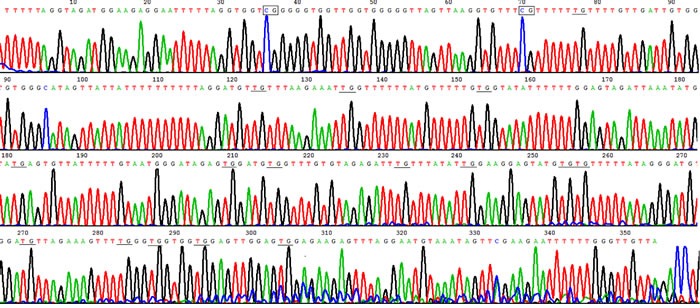
Hypomethylation in HLA-DRB1 promoter in psoriatic lesional epidermis Example of sodium bisulfite genomic sequencing product in psoriatic lesion showed HLA-DRB1 methylation only occurred in 2 out of 18 CpG sites. The rectangles show methylated CpG, and the underline show unmethylated CpG where C change to T after bisulphate treatment.

**Figure 3:**
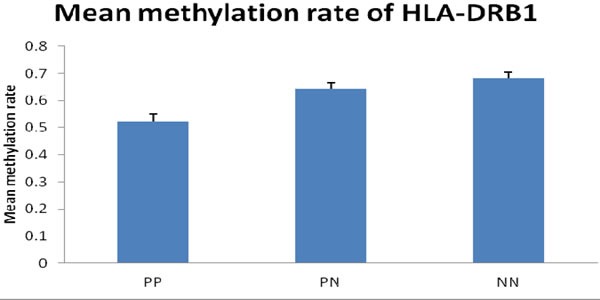
Mean methylation rate of HLA-DRB1 in psoriatic skin (*n* = 56) and healthy controls (*n* = 28) The frequency of promoter methylation for HLA-DRB1 in PP was significantly lower than in PN (*t* = 13.077, *p* < 0.001), and there is no significant difference between the rate of promoter methylation in PN and NN. PP: psoriatic lesion, PN: psoriatic non-lesion, NN: healthy controls

**Figure 4:**
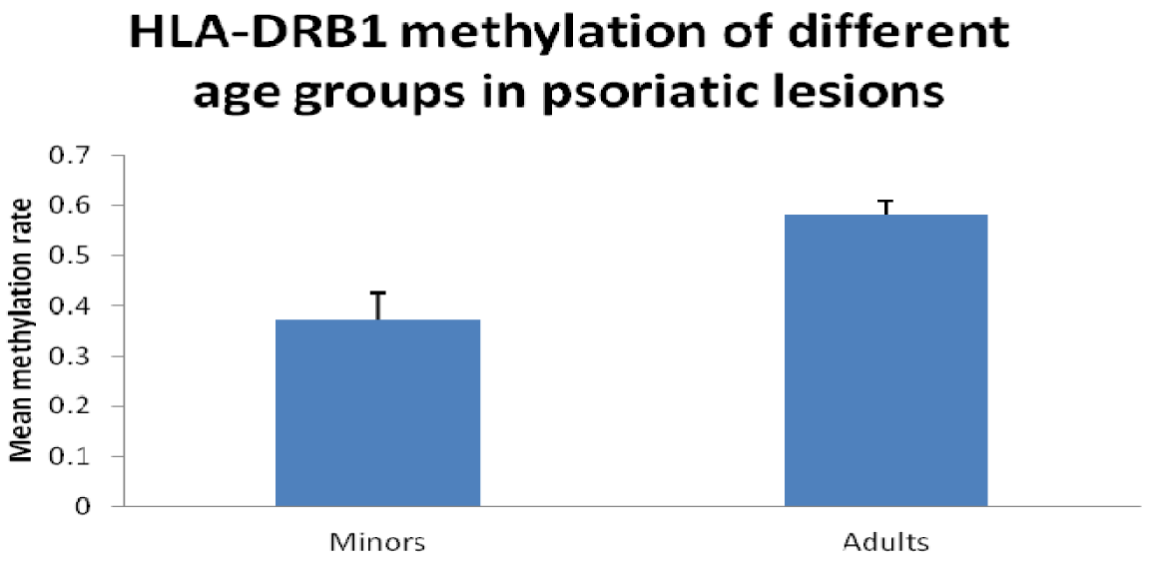
HLA-DRB1 methylation of different age groups in psoriatic lesions Mean methylation rate of HLA-DRB1 of the minors group with onset age≤18 years is significantly lower than the adults group with onset age > 18 years in psoriatic lesions (37.4±20.4% and 58.2±16.5%; *t* =3.968, *p* < 0.001).

**Figure 5:**
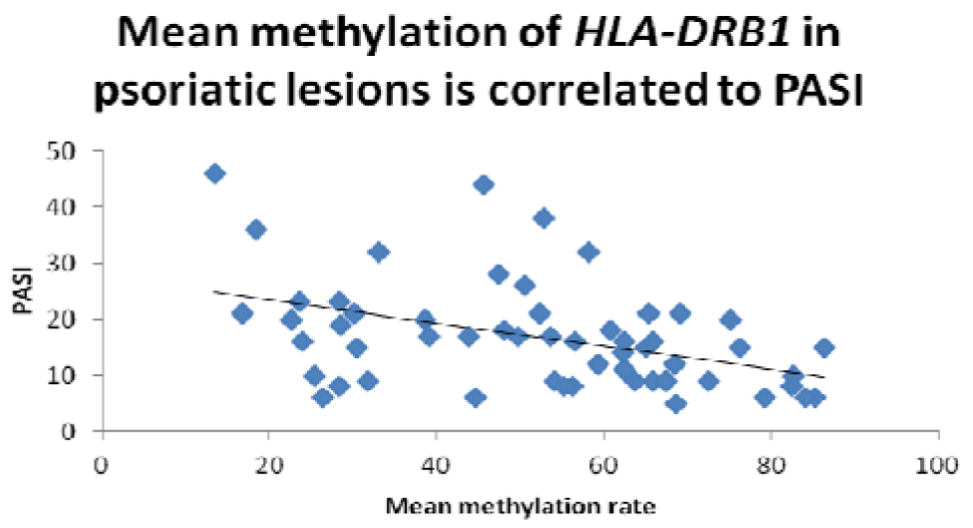
The mean methylation rate of HLA-DRB1 in psoriatic lesions is negatively correlated to PASI score (*r* = -0.431, *p*=0.001)

First, we performed bisulfite sequencing PCR (BSP) analysis to determine the methylation status in the promoter regions of HLA-DRB1 in epidermis of psoriatic lesions, psoriatic non-lesions and healthy volunteers. Methylation of HLA-DRB1 was found in all samples, and bisulfite sequencing results indicated that 3-18 of 18 CpG dinucleotides in the promoter region of the HLA-DRB1 were methylated (Figure 1 and 2). In psoriatic lesions, psoriatic non-lesions and healthy controls, the mean rate of promoter methylation for HLA-DRB1 was 52.26±2.66%, 64.34±2.30% and 68.11±2.18%, respectively. The frequency of promoter methylation for HLA-DRB1 in psoriatic lesions was significantly lower than in psoriatic non-lesions (*t* = 13.077, *p* < 0.001), and there is no significant difference for the rate of HLA-DRB1 methylation between in psoriatic non-lesions and healthy controls (*t* = 1.046, *p* = 0.299) (Figure 3). The mean methylation rate of HLA-DRB1 of the minors group with onset age≤18 years is significantly lower than the adults group with onset age >18 years in psoriatic lesions (37.43±5.10% and 58.19±2.61%; *t* = 3.968, *p* < 0.001) (Figure 4). The mean methylation rate of HLA-DRB1 in psoriatic lesions is negatively correlated to PASI score (*r* = -0.431, *p* = 0.001) (Figure 5).

### HLA-DRB1 mRNA expression

**Figure 6:**
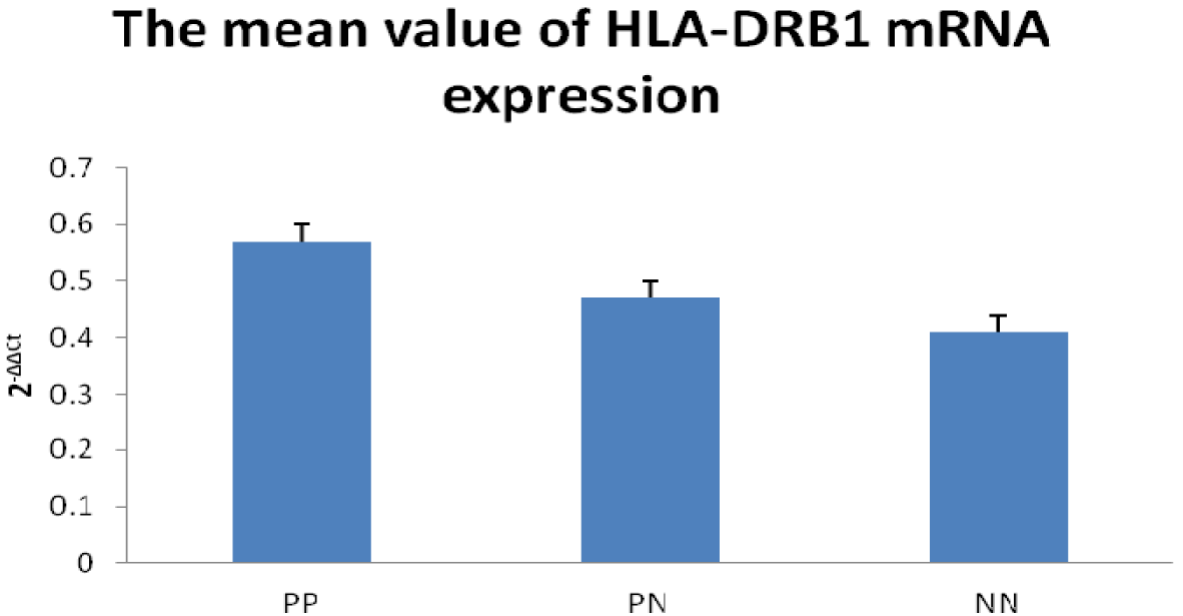
Realtime RT-PCR to detect for HLA-DRB1 mRNA expression The mean value of HLA-DRB1 mRNA expression (2-ΔΔCt) was significantly higher in PP than PN, while there is no significant difference between PN and NN. PP: psoriatic lesion, PN: psoriatic non-lesion, NN: healthy controls

**Figure 7:**
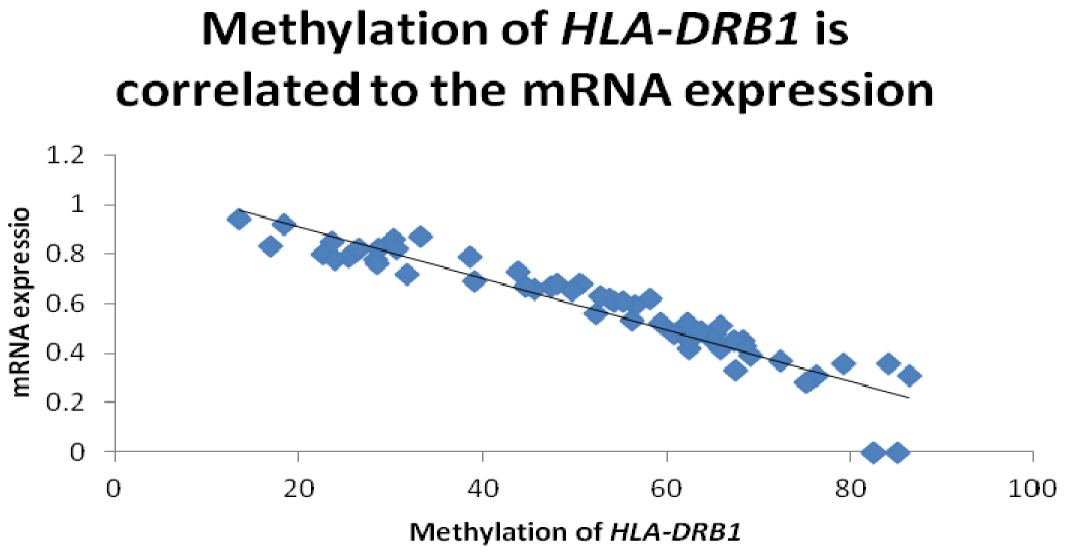
The promoter methylation of HLA-DRB1 is negatively correlated to the mean value of HLA-DRB1 mRNA expression in psoriatic lesions(*r* = -0.932, *p* < 0.001)

In order to examine whether HLA-DRB1 methylation can affect the level of HLA-DRB1 expression in psoriasis, we performed RT-PCR analysis to determine the level of HLA-DRB1 mRNA in the specimens of epidermis. We have observed the expression of HLA-DRB1 in 94.6% (53/56) psoriatic lesions, 92.9% (52/58) psoriatic non-lesions and 92.9% (26/28) epidermis of healthy controls. The mean mRNA expression of HLA-DRB1 in psoriatic lesions, psoriatic non-lesions and healthy controls is 0.57±0.03, 0.47±0.03 and 0.41±0.03, respectively (Figure 6). HLA-DRB1 mRNA expression in psoriatic lesions was higher than psoriatic non-lesions (*t* = 12.119, *p* < 0.001). There are no significant difference for the expression of HLA-DRB1 mRNA between in psoriatic non-lesions and healthy controls (*t* = 1.172, *p* = 0.245). The mean value of HLA-DRB1 mRNA expression is negatively correlated to the methylation of HLA-DRB1 in psoriatic lesions(*r* = -0.932, *p* < 0.001) (Figure 7).

## DISCUSSION

DNA methylation is an important epigenetic mechanism that can affect gene expression without changing the DNA sequence. Some recent studies indicated that aberrant methylation status of HLA genes may be involved in the pathogenesis of some diseases. The abnormal methylation of HLA-G gene has been found in cancers [24, 25] and preeclampsia [26]; and hypermethylation of HLA-A, B, C was observed in esophageal squamous cell carcinoma (ESCC) [27]. For HLA-DRB1, Hu et al. proved that HLA-DRB1B1 methylation changes can promote the occurrence and progression of ESCC [28]; Yu et al. reported the association between brain DNA methylation of HLA-DRB1B5 and pathological diagnosis of Alzheimer disease [29]; Graves et al. discovered that methylation at the HLA-DRB1B1 locus in CD4+ T cells are associated with multiple sclerosis [30].

In our study, we first reported the hypomethylation of HLA-DRB1 in psoriatic epidermis; more importantly, our data proved that methylation of HLA-DRB1 is negatively correlated with the severity of the disease, suggesting that the methylation status of HLA-DRB1 can become a potential bio-marker for the diagnosis or evaluating the efficacy of treatment of psoriasis. Interestingly, previous work has also correlated aberrant methylation of HLA-DRB1 with the prognosis of some diseases, thus, we hypothesis that methylation status of HLA-DRB1 may also affect the prognosis and recurrence of psoriasis, which can be proposed as our future direction for the investigation of HLA-DRB1 methylation in psoriasis.

Roberson et al. [31] found that the frequency of methylation was strongly associated with gene expression at a nearby locus at only 12/1,100 CpG sites, suggesting that the level of methylation may only affect the expression of a small size of genes. The promoter and 3’-UTR of HLA-DRB1 can antagonistically regulate the gene expression [32]. In order to explore if HLA-DRB1 promoter hypomethylation can directly affect the expression of HLA-DRB1, we also examined HLA-DRB1 mRNA expression in all epidermis from psoriatic lesions, psoriatic non-lesions and healthy controls. The expression of HLA-DRB1 mRNA was significantly increased in psoriatic epidermis when compared with the healthy volunteers, and the mean value of HLA-DRB1 mRNA expression is negatively correlated to the promoter methylation of HLA-DRB1. Our results indicated that hypomethylation of HLA-DRB1 may be one of the leading causes that induced the overexpression of HLA-DRB1 in psoriatic epidermis, which may promote the development of the disease.

The impact of the hypomethylation and overexpression of HLA-DRB1 on keratinocytes is still unclear. It was demonstrated that several peptides on keratin 17 can serve as immunodominant T-cell epitopes and stimulate peripheral blood lymphocytes of HLA DRB1*04 and/or *07 positive patients with psoriasis. Moreover, the peptide ligands are able to inhibit proliferation of T cells and keratinocyte in psoriasis [33].

One limitation of present study is that we only performed analysis of HLA-DRB1 expression on mRNA level for the limited epidermal specimens, and the expression of HLA-DRB1 protein in samples have not been examined. For every patient, only a small piece of epidermis could be collected, thus the samples were not enough for both nucleic acid level and protein level analysis. To investigate the specific role of hypomethylation of HLA-DRB1 in the pathogenesis of psoriasis, in the following step, we plan to perform animal studies using the existing psoratic models.

To the best of our knowledge, this is the first paper reporting an association between methylation of HLA-DRB1 gene and psoriasis. Overall, abnormal hypomethylation of HLA-DRB1 gene promoter has been observed in psoriatic epidermis. Hypomethylation of HLA-DRB1 associated with the mRNA level of HLA-DRB1 expression and severity of the disease, indicating that hypomethylation of HLA-DRB1 may also play a potential role in the pathogenesis of psoriasis.

## MATERIALS AND METHODS

### Subjects and skin samples

Skin samples were collected from 56 patients with active plaque psoriasis (21 females and 35 males) and 28 healthy volunteers. Their characteristics are summarized in Table 1. A paired psoriatic lesion (PP) and psoriatic non-lesion (PN) were derived from the same patient, thus a total number of 56 paired PP/PN samples were obtained. The psoriatic non-lesion specimens were taken from the sites more than 5 cm far away lesion edge. The patients were between 18 to 65 years old (mean 37.25±1.68), with the length of disease ranged from 1 to 33 years (mean 7.41±0.99). Among all 56 patients, the onset age of 40 cases was more than 18 years old. The severity of the disease was evaluated using the psoriasis area severity index (PASI) scoring system and the score was ranged from 4 to 46 (mean 16.78±1.27). Patients that received either systemic or topical therapy 6 weeks prior to this study and pregnant patients were excluded. Healthy volunteers (NN) were people who did not have skin lesions and without self-reported psoriasis history. The NN group and PP/PN group has a qualified match in age and sex. All tissue specimens were obtained following the ethical guidelines mandated by the ethical committee of Institute of Dermatology, Chinese Academy of Medical Sciences who approved this study. All candidates were approached using approved ethical guidelines and those who agreed to participate in this study were required to sign the consent forms. Tissue specimens on trunk or limbs were obtained using a 6-mm punch biopsy under aseptic conditions with local anesthesia. The specimens were frozen in liquid nitrogen and stored at −80 °C until processed.

**Table 1:** Demographics and disease parameters of patients with psoriasis

Case	Gender	Age (y)	Length of disease (y)	Onset age(y)	PASI	Family history	Methy in lesions%	Methy in non-lesions%	Expression in lesions	Expression in non-lesions
1	M	32	1	31	21	-	52.3	71.2	0.56	0.43
2	M	46	20	26	32	-	58.2	74.5	0.62	0.51
3	M	45	10	35	16	-	56.5	67.8	0.59	0.48
4	M	28	8	20	9	-	65.9	83.3	0.42	0.35
5	M	65	8	57	44	-	45.6	57.6	0.66	0.49
6	M	30	6	24	12	-	68.4	88.5	0.45	0.37
7	F	30	14	16	19	+	28.5	54.3	0.82	0.73
8	M	48	1	47	21	-	69.2	76.6	0.39	0.32
9	F	52	3	49	15	-	65.1	82.4	0.45	0.38
10	M	21	3	18	11	-	62.5	59.1	0.42	0.34
11	F	46	3	43	15	-	86.4	86.2	0.31	0.23
12	M	36	10	26	9	+	31.7	48.7	0.72	0.58
13	F	33	20	13	21	+	16.8	39.1	0.83	0.69
14	M	18	1	17	6	+	44.6	48.5	0.67	0.53
15	F	38	20	18	17	-	53.7	62.2	0.62	0.49
16	F	19	2	17	10	+	25.4	42.5	0.79	0.65
17	M	48	10	38	9	-	54.2	65.6	0.61	0.65
18	F	52	20	32	21	-	65.3	75.1	0.48	0.32
19	M	43	1	42	10	-	82.6	86.4	0	0
20	M	35	6	29	23	-	28.3	48.2	0.78	0.72
21	M	20	5	15	36	+	18.4	42.6	0.92	0.78
22	F	29	1	28	9	-	67.5	79.4	0.33	0.25
23	M	22	4	18	23	+	23.6	48.5	0.85	0.78
24	M	32	8	24	6	-	79.2	84.1	0.36	0.27
25	M	36	5	29	26	-	50.6	72.3	0.68	0.64
26	M	58	16	42	28	-	47.5	72.1	0.67	0.61
27	M	64	1	63	20	-	38.6	49.2	0.79	0.72
28	F	34	16	18	8	-	56.2	55.4	0.53	0.37
29	M	17	5	12	46	+	13.5	32.6	0.94	0.86
30	M	49	15	34	38	-	52.8	67.7	0.63	0.58
31	F	22	3	19	6	+	26.4	36.8	0.82	0.65
32	F	55	33	22	21	+	30.2	41.6	0.86	0.73
33	F	28	1	27	17	-	49.7	59.3	0.65	0.57
34	F	40	3	37	17	-	39.1	42.9	0.69	0.61
35	M	39	30	9	8	+	28.4	39.2	0.76	0.65
36	M	22	5	17	20	+	22.6	38.6	0.80	0.72
37	M	43	1	42	6	-	85.2	86.5	0	0
38	F	23	10	13	9	-	72.5	84.8	0.37	0
39	M	52	17	35	15	-	76.3	86.6	0.31	0.22
40	M	39	1	38	5	-	68.6	78.2	0.43	0.25
41	F	18	1	17	16	+	23.9	38.6	0.78	0.65
42	M	46	2	44	14	-	62.3	72.7	0.52	0.43
43	F	21	1	20	8	-	82.5	85.3	0	0
44	F	23	7	16	20	-	75.2	88.4	0.28	0.26
45	M	44	11	33	17	-	43.8	47.5	0.73	0.62
46	M	48	5	43	18	-	48.1	60.8	0.68	0.59
47	M	52	3	49	8	-	55.2	75.6	0.61	0.42
48	F	46	2	44	9	-	67.4	81.2	0.45	0.28
49	M	29	5	24	12	-	59.3	61.3	0.52	0.37
50	M	53	5	48	18	-	60.8	70.6	0.48	0.32
51	M	32	1	31	16	-	62.5	74.8	0.45	0.35
52	M	33	8	25	16	-	65.9	76.2	0.51	0.39
53	F	26	8	18	32	+	33.1	45.5	0.87	0.85
54	F	32	2	30	9	-	63.7	75.1	0.49	0.26
55	M	43	6	37	15	+	30.5	44.5	0.82	0.76
56	F	51	1	50	6	-	84.2	88.5	0.36	0.18
Mean		37.25	7.41	29.8	16.77		52.3	64.3	0.59	0.49

### Epidermal isolation, DNA and RNA extraction

Epidermis were separated from skin specimens using 0.25% Dispase (Roche, USA) for 6-12 hours; next, the samples were placed on ice, and homogenized in sterilized 5-mL tissue grinders; then, DNA and total RNA were extracted from the epidermis samples using QIAamp DNA Mini kit and RNA Mini kit (Qiagen, Valentia, CA) following the manufacturer's protocols. The quality of the extracted total RNA were assessed using electrophoresis on 1% agarose gels, by visualizing the 18S and 28S RNA bands under UV light.

### Bisulphite conversion

Bisulphite conversion of genomic DNA was performed was performed using EpiTectH Bisulfite kit (Qiagen) following the manufacturer's instructions. Briefly, genomic DNA samples (50 ~ 150 ng DNA/sample) were denatured using NaOH, and then treated with sodium bisulfite; next, the samples were desulfonated,and purified using silica-membrane columns; finally, the treated DNAs were suspended in 10μl TE buffer either for immediate measurement, or stored at -20 °C for future analysis.

### Sodium bisulfite genomic sequencing PCR

To investigate the methylation status of the HLA-DRB1 promoter, we mapped the corresponding CpG island in HLA-DRB1 by CpGplot (EBI Tools, EMBOSS CpGPlot; http://www.ebi.ac.uk/emboss/cpgplot). CpG-enriched region of HLA-DRB1 was amplified using primers as follow: F-5’- TTTTAGGTAGATGGAAGAGGAA -3’ and R-5’- AACAACCCAAAAAATTCTTCTA -3’ (352bp). Modified DNAs were used for PCR analysis, and the products include 18 CpG sites and span part of the promoter of HLA-DRB1. The reaction was set up at a total volume of 25 ml containing 1 ml sample DNA, 0.2 mM dNTPs, 0.5 mM primers, 2.5 mM MgCl2, 16buffer II, and 1.25 unit HotTaq DNA polymerase (Takara, JP).The thermocycle profiles was 94 °C for 9 minutes to activate the polymerase; 44 cycles at 94 °C for 30 seconds, 55 °C for 30 seconds, and 68 °C for 45 seconds; and a 7-minute terminal extension at 68 °C. The amplified DNA fragments were then cloned into plasmids using a TOPO TA Cloning kit (Invitrogen, Carlsbad, CA) according to manufacturer's instructions .The clones were then screened by colony PCR, and purified using the Qiaquick kit (Qiagen, CA). Next, individual clones were sequenced on an ABI PRISM 3730 automated sequencer, and the sequence electropherograms were aligned using Sequence Navigator software (Applied Biosystems). Quantitative methylation rates were estimated from sequence traces using the ESME software.

### Real-time RT-PCR analysis for mRNA expression

Total RNA extracted from epidermis was converted to amplified cDNA using a GoScript™ Reverse Tanscription System Kit (Promega, USA) on the DNA Engine Opticon System (ABI GeneAmp PCR System 7300, USA), and cDNAs were aliquoted and stored at -20 °C. In brief, the cDNA synthesis contained 5 uL of 10× Taqman RT buffer; 11 uL of 25 mM MgCl2; 10 uL of deoxy NTPs; 2.5 uL of Oligo d(T)16 primer; 1 uL of RNase inhibitor; 1.25uL of Multiscribe reverse transcriptase, and 1 ug of RNA. Two separate 50 uL reactions for each RNA were performed and combined together. Each cDNA batch reaction had a maximum of 24 tubes to ensure the best sample quality. Primers were as follow: HLA-DRB1 F- 5’- TGCCAAGTGGAGCACCCAA -3’ and R-5’- GCATCTTGCTCTGTGCAGAT -3’; β-ACTIN F-5’- CAGTCGGTTGGAGCGAGCAT -3’ and R-5’- GGACTTCCTGTAACAACGCATCT -3’. The primer test results showed that all cDNA amplified with a single band. This detection ensured that the primers were specific to HLA-DRB1. Quantitative PCR (qPCR) was performed on an ABI 7900HT Sequence Detection System (Applied Biosystems) in 384-well plates. The samples were run in triplicate using one plate per gene. The reaction was performed in a 12.5 uL total volume with 6.5 uL of 2× SYBR Green Master Mix (Applied Biosystems); 0.25 uL of 10 uM forward primer; 0.25 uL of 10 uM reverse primer; 4uL of a 1:10 dilution of cDNA template (corresponding to approximately 8 ng RNA). The thermal cycle conditions were: 50 °C for 2 min, 95 °C for 10 min, 45 cycles at 95 °C for 15 s, and 62 °C for 1 min, and a final dissociation step at 95 °C for 15 s, 65 °C for 15 s, and 95 °C for 15 s. The qPCR cycle threshold (Ct) was set in the middle of the exponential phase of the amplification. Also, appropriate positive and negative controls were included in each run. In each experiment, the individual sample was run in triplicate and the Ct of each well was recorded at the end of the reaction. The mean and standard deviation (SD) of the three Cts were calculated and the average value was accepted if the triplicate Ct values were within±1 Ct.

For each sample, HLA-DRB1 expression was presented as the ratio between HLA-DRB1 and the endogenous control (β-ACTIN) using the 2−ΔΔCt method [34]. The identity of polymerase chain reaction (PCR) products was verified by sequencing.

### Statistical analysis

SPSS 17.0 was used for all statistic analyses. The methylation and RT-PCR results are presented as mean ± SE. The t-test was used to compare the difference between paired and unpaired observations. Adjusted odds ratios (ORs) were calculated by fitting logistic regression models with adjustment. *P*-values < 0.05 were considered statistically significant.
